# Diagnostic accuracy of risk assessment and fecal immunochemical test in colorectal cancer screening: Results from a population‐based program and meta‐analysis

**DOI:** 10.1002/cam4.6399

**Published:** 2023-08-14

**Authors:** Ziyang Wang, Jiaoyue Teng, Weimiao Wu, Jianming Dou, Martin C. S. Wong, Yangming Gong, Junjie Huang, Kai Gu, Wanghong Xu

**Affiliations:** ^1^ Department of Epidemiology Fudan University School of Public Health Shanghai China; ^2^ Yiwu Research Institute Fudan University Yiwu China; ^3^ The Shanghai Municipal Centers for Disease Control and Prevention Shanghai China; ^4^ Jockey Club School of Public Health and Primary Care, Faculty of Medicine The Chinese University of Hong Kong Hong Kong China

**Keywords:** colorectal cancer, diagnostic accuracy, fecal immunochemical test, risk assessment, screening

## Abstract

**Background:**

Fecal immunochemical test (FIT) is a commonly used initial test for colorectal cancer (CRC) screening. Parallel use of FIT with risk assessment (RA) could improve the detection of non‐bleeding lesions, but at the expense of compromising sensitivity. In this study, we evaluated the accuracy of FIT and/or RA in the Shanghai CRC screening program, and systematically reviewed the relevant evaluations worldwide.

**Methods:**

RA and 2‐specimen FIT were used in parallel in the Shanghai screening program, followed by a colonoscopy among those with positive results. Sensitivity, specificity, detection rate of CRC, positive predictive value (PPV), and other measures with their 95% confident intervals were calculated for each type of tests and several assumed combined tests. We further searched PubMed, Embase, Web of Science, and Cochrane Library for relevant studies published in English up to January 5, 2022.

**Results:**

By the end of 2019, a total of 1,901,360 participants of the screening program completed 3,045,108 tests, with 1,901,360 first‐time tests and 1,143,748 subsequent tests. Parallel use of RA and 2‐specimen FIT achieved a sensitivity of 0.78 (0.77–0.80), a specificity of 0.78 (0.78–0.78), PPV of 0.89% (0.86–0.92), and a detection rate of 1.99 (1.93–2.05) for CRC per 1000 among participants enrolled in the first screening round, and performed similarly among those who participated for several times. A meta‐analysis of 103 published observational studies demonstrated a higher sensitivity [0.76 (0.36, 0.94)] but a much lower specificity [0.59 (0.28, 0.85)] of parallel use of RA and FIT for detecting CRC in average‐risk populations than in our subjects. One‐specimen FIT, the most commonly used initial test, had a pooled specificity comparable to the Shanghai screening program (0.92 vs. 0.91), but a much higher pooled sensitivity (0.76 vs. 0.57).

**Conclusion:**

Our results indicate the limitation of FIT only as an initial screening test for CRC in Chinese populations, and highlight the higher sensitivity of parallel use of RA and FIT. Attempts should be made to optimize RA to improve effectiveness of screening in the populations.

## 1 BACKGROUND

Colorectal cancer (CRC) is the third common cancer globally.^1^ Early detection of CRC by conducting large‐scale screening programs has reduced the incidence and mortality of the cancer around the world.^2^ Colonoscopy, as a gold standard for CRC screening, has been used as a primary screening test in developed countries. In China and other low‐ or middle‐income countries, however, it is not feasible to apply this one‐step strategy due to the large populations, insufficient health resources, and limited colonoscopy capacities.^3^ Instead, a triage screening is currently implemented using questionnaire‐based risk assessment (RA), guaiac fecal occult blood test (gFOBT), fecal immunochemical test (FIT), flexible sigmoidoscopy (FS), or computed tomography colonography alone or combined to identify high‐risk individuals for subsequent colonoscopy assessment.^4^, ^5^, ^6^ In the context, the accuracy of the initial tests is essential for a successful CRC screening program.

Of the aforementioned initial screening tests, FIT is most widely used for its high sensitivity, noninvasive nature, simplicity, and convenience.^7^ Questionnaire‐based RA, on the other hand, was usually used with other tests to reduce the workload of colonoscopy, particularly among resource‐limited settings.^8^ Parallel use of RA and FIT has been found to improve the detection of non‐bleeding lesions in Chinese populations, but at the expense of compromising sensitivity.^9^ Huang et al.^10^ found that compared with FOBT only, parallel use of FOBT and RA was more cost‐effective among populations in urban China. In our previous reports based on the Shanghai CRC screening program, we optimized the risk scoring systems and improved the performance of parallel use of RA and FIT in detecting CRC and colorectal adenomas.^11^, ^12^ The accumulating supportive evidence has promoted the recommendation of parallel use of RA and FIT for CRC screening in China.^13^

In this study, we evaluated the accuracy of FIT and/or RA in detecting CRC among participants of the Shanghai CRC screening program. We also compared the sensitivity, specificity, positive predictive value (PPV), detection rate of CRC and other indicators of the tests in our subjects with other populations through a meta‐analysis. Our results may inform the selection of initial screening tests, and provide evidence for policy making for CRC screening in Chinese populations and other resource‐limited settings.

## 2 MATERIALS AND METHODS

### 2.1 Study design and population

As described previously, the Shanghai CRC screening program was initiated in 2013 as a key public health service by the Shanghai municipal government.^14^ The program was designed as a long‐term multiple‐wave screening service with specific numbers of participants for each 3‐year wave of screening. The screening tasks were assigned to each district and then to each community health center (CHC). All permanent residents and migrant populations aged 50–74 years having no history of CRC were eligible for the program and were mobilized by local CHC through posters, phone calls, and home visits until achieving the planned number of participants. Re‐invitation was not permitted within one year of screening but was encouraged in different waves.

**FIGURE 1:**
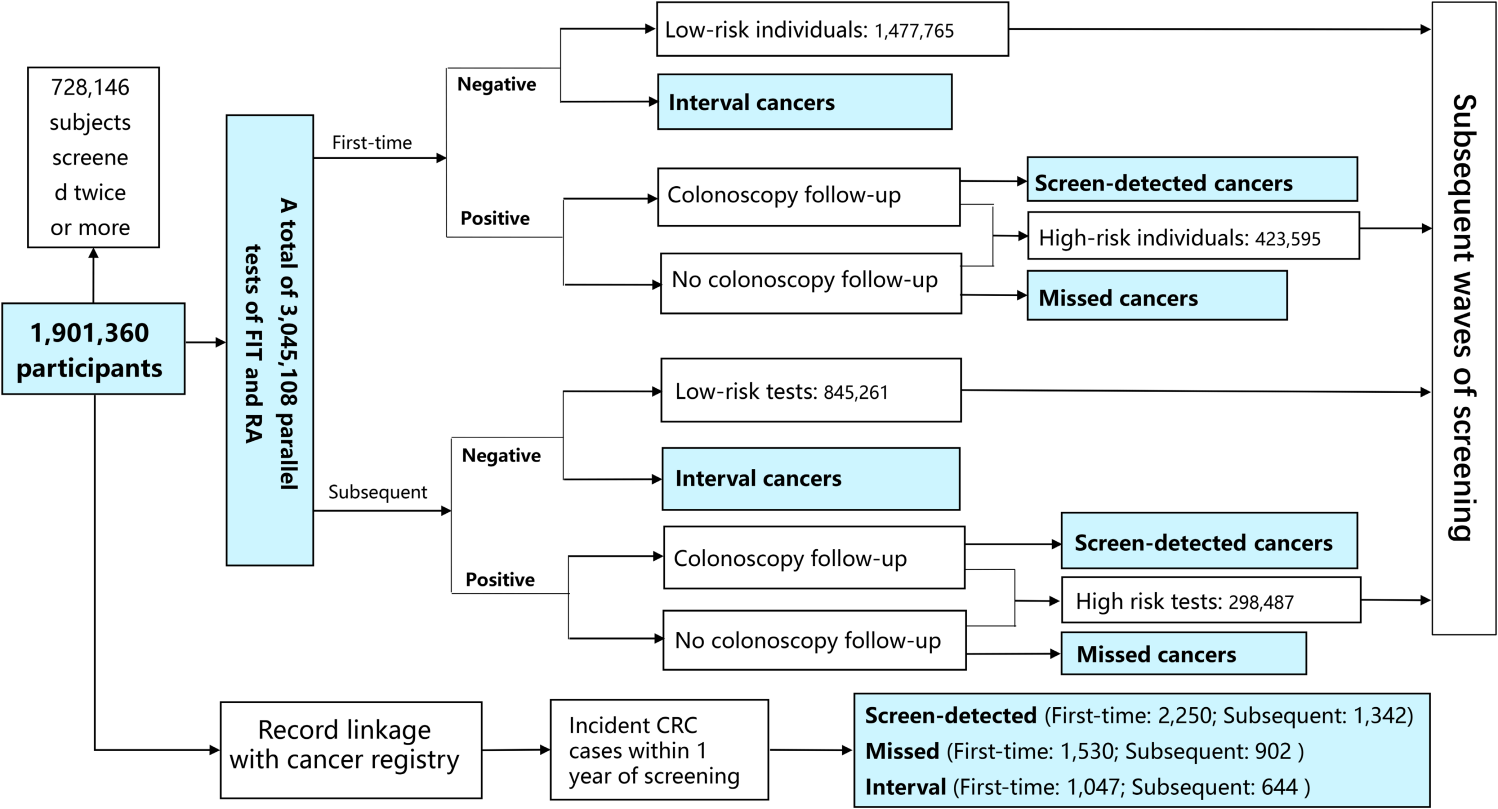
Screening modality of the Shanghai CRC screening program. Screen‐detected cancers defined as those diagnosed within 90 days of screening; supplemented cancers included missed and interval cancers diagnosed within 2 years of screening with positive or negative results in initial tests. FIT, fecal immunochemical test; RA, risk assessment.

All subjects were informed of potential benefits and risks of the screening, and were required to provide signed consent before participation. People who were not eligible but asked to participate in the program also received the screening but were excluded from this analysis. A total of 1,901,360 eligible local residents were screened during the period of 2013–2019, and 728,146 subjects participated in the program more than once (Figure 1).

### 2.2 Data collection

All participants completed questionnaire‐based RA and 2‐specimen qualitative FIT as initial screening. Briefly, a structured questionnaire was used to collect information on demographic characteristics (date of birth, sex, educational level, marital status, occupation, and area of residence), and the responses to nine questions for risk stratification. As reported previously,^9^ the subjects were regarded as high‐risk for CRC if they fulfilled one of the three conditions: (1) diagnosis of any cancer; (2) history of colorectal polyps; (3) CRC in first‐degree relatives, and/or at least two of the six conditions: (1) history of chronic constipation; (2) history of chronic diarrhea; (3) history of mucus or bloody stool; (4) serious unhappy life events that caused psychiatric trauma (e.g., divorce, death of any family member); (5) chronic appendicitis or appendectomy within 2 years; (6) chronic cholecystitis or cholecystectomy within 2 years. The RA questionnaire was developed and validated in previous studies,^9^, ^15^ and recommended in the 2006 guideline for CRC screening in China. All staff of this program were previously trained with the protocol of the program. The results of RA were double‐checked by the district centers for disease control and prevention (CDC) per month to make sure high quality of the data.

After completing the questionnaire, all participants were instructed to collect two moist fecal specimens in two separate 5 mL tubes within an interval of 7 days, and were required to return the specimens to a community health center nearby within 48 h. The minimal detection of FIT was 100 ng Hb/mL (20 μg Hb/g feces). Participants having a positive result in either RA or FIT were identified as high‐risk individuals, and were referred to officially designated or self‐selected hospitals for colonoscopy examination of the entire colon up to the ileocecus, if applicable.

The test results were entered into a reporting system, based on which the screen‐detected CRC, that is, subjects positive in initial screening and diagnosed with CRC within 90 days of screening, could be obtained. The potentially missed or interval cases were supplemented through a record linkage with the Shanghai Cancer Registry using a unique ID number. The missed cases were defined as those who had a positive result in initial screening but were diagnosed with CRC after 90 days but within 1 year of screening, while the interval cases referred to those negative in initial screening but diagnosed with CRC within 1 year of screening.

### 2.3 Statistical analysis

In addition to the currently used initial test in the program, that is, parallel use of RA and 2‐specimen FIT, we assumed to apply RA only, first FIT only, 2‐specimen FIT only, parallel use of RA and first FIT, serial use of RA and first FIT, or serial use of RA and 2‐specimen FIT as the initial tests. The positive result for parallel test of RA and first FIT was defined as either RA or FIT of the first fecal specimen was positive, while that for serial test of RA and first FIT referred to positive results of both RA and FIT of the first fecal specimen. Serial use of RA and 2‐specimen FIT considered individuals with positive results in RA and both FIT as at high risk of CRC. Then we calculated the sensitivity, specificity, PPV, positive likelihood ratios (LR^+^), negative likelihood ratios (LR^−^), areas under the receiver operating characteristic curve (AUC), and detection rates of CRC of the currently used and assumed initial tests for comparisons. Considering the probably different performance of these tests in subjects attending the screening for the first time and for multiple times, we estimated the indices among the two settings, respectively.

### 2.4 Meta‐analysis

The systematic review followed a registered protocol (PROSPERO CRD42022316074), and was reported in accordance with the Reporting Checklist for Meta‐analyses of Observational Studies (MOOSE) and Preferred Reporting Items for Systematic Reviews and Meta‐Analyses (PRISMA) guidance.

We searched PubMed, Embase, Web of Science, and Cochrane Library from their inception to January 5, 2022 for observational studies published in English, limiting to those using FIT and/or RA as the initial screening tests for CRC. The detailed search strategy is shown in Table S1. The electronic database searches were supplemented with manual searches of the reference lists of the articles included.

The search results were retrieved to an EndNote X9 database, and the duplicates were removed. The inclusion criteria included: (1) CRC screening was conducted in an average‐risk population; (2) RA and FIT were used alone or combined as initial screening tests for CRC; (3) the confirmation of CRC was based on pathological results; (4) data were available for calculation of sensitivity, specificity, PPV, LR^+^, LR^−^, AUC, and detection rate of CRC of initial screening tests. The studies excluded were: (1) reviews, case reports, editorials, commentaries, letters, guidelines, and conference abstracts due to their insufficient information for assessment; (2) reports on outcomes of colorectal neoplasia without distinguishing the CRC from other colorectal lesions.

Two independent authors (Z.Y.W. and J.Y.T.) assessed the titles, abstracts, full texts, and reference lists of relevant publications according to the predefined eligibility criteria for final inclusion. They also independently extracted the following data from the selected studies: authors, year of publication, country/region where the study was conducted, study design, study period, total number of participants, recruitment method, waves of screening, residence of subjects, initial screening tests, positivity thresholds of FIT, true positives, true negatives, false positives, and false negatives. A standardized electronic database was developed with the extracted data above. The quality and applicability of the included studies were assessed by the authors using the Quality Assessment of Diagnostic Accuracy Studies‐2 (QUADAS‐2) tool.^16^ Any disagreements in the study selection, data extraction, and quality assessment were resolved by discussion with the third reviewer (W.M.W.) to achieve a consensus.

Sensitivity, specificity, PPV, LR^+^, LR^−^, AUC, detection rate of CRC and their corresponding 95% confidence intervals (CIs) were computed for each included study. Pooled estimates of sensitivity and specificity were calculated through a bivariate diagnostic random effects meta‐analysis using the riley function of the R package “metamisc.”^17^, ^18^ Pooled LR^+^, LR^−^_,_ and corresponding 95% CIs were estimated using bivariate model according to the method reported by Zwinderman and Bossuyt^19^ The summary receiver operating characteristics curves (sROC) were plotted with weighing by sample size of included studies, with the AUC applied to estimate the discriminative ability of each screening test. The detection rate of CRC were pooled using R package “meta,” with metaprop function used to estimate potential missed cases.^20^ *I* ^2^ was estimated using Meta_DiSc software,^21^ and used to assess statistical heterogeneity for the bivariate meta‐analysis.^22^ Stratified meta‐analyses and meta‐regression analyses were further conducted to explore the potential sources of heterogeneity, including type of screening (organized/opportunistic), type of initial test (RA only, FIT only, parallel of RA and FIT, serial of RA and FIT), number of FIT used (1‐specimen /2‐specimen), type of FIT (qualitative or quantitative), and threshold for positive FIT. We also estimated the posttest probability of negative or positive result for FIT only or parallel test of FIT and RA in the meta‐analysis based on the pretest probability of 0.01 (namely previously reported CRC prevalence),^23^ as well as the pooled LR^+^ and LR^−^, based on which we illustrated the posttest probability using Fagan's nomogram.^24^ Publication bias was evaluated by means of Deek's funnel plot asymmetry test.^25^

We further performed a sensitivity analysis by excluding the studies with poor quality (high risk of bias as measured by QUADAS‐2) and those with less than 1000 participants. We also compared the methods and the results of this meta‐analysis with those in previous systematic reviews.

## 3 RESULTS

### 3.1 The Shanghai CRC screening program

A total of 798,488 men and 1,102,872 women completed both RA and 2‐specimen FIT for the first time during 2013–2019, covering around one fourth of the population aged 50–74 in Shanghai. Of the 1,901,360 participants, 423,595 had a positive result in initial screening, with 162,476 (8.5%) being positive only in RA, 225,751 (11.9%) being positive only in FIT, and 35,368 (1.9%) being positive in both tests. As a result, a total of 4827 CRC cases were identified, with 2250 detected by screening and 2577 (1530 missed and 1047 interval) supplemented through a record linkage with the Shanghai Cancer Registry (Table 1). Among 728,146 subjects participating in the program twice or more, a total of 1,143,748 additional initial tests were performed, leading to 298,487 tests with a positive result, in which 131,716 (11.5%) were positive only in RA, 137,315 (12.0%) positive only in FIT, and 29,456 (2.6%) positive in both tests. Of the 2888 CRC cases identified, 1342 were screen‐detected, 902 were missed cases, and 644 were interval cancers.

**TABLE 1:** Detected CRC in participants of the Shanghai CRC screening program.

	Initial screening		Number of CRC		
	Subjects/Tests	Positive, *N* (%)	Screen‐detected, *N* (%)	Missed, *N* (%)	Interval, *N* (%)
Among first‐time participants (*n* = 1,901,360)					
All subjects	1,901,360	423,595 (22.3)	2250 (44.6)	1530 (31.7)	1047 (21.7)
Sex					
Men	798,488	181,061 (22.7)	1254 (45.9)	890 (32.6)	586 (21.5)
Women	1,102,872	242,534 (22.0)	996 (47.5)	640 (30.5)	461 (22.0)
Age (years)					
50–59	664,535	138,056 (20.8)	475 (49.1)	308 (31.8)	185 (19.1)
60–69	1,001,683	228,964 (22.9)	1357 (46.7)	906 (31.2)	644 (22.2)
70–74	235,142	56,575 (24.1)	418 (43.9)	316 (33.2)	218 (22.9)
Initial test					
RA positive only	‐	162,476 (8.5)	188 (45.4)	226 (54.6)	
FIT positive only	‐	225,751 (11.9)	1653 (60.4)	1083 (39.6)	
Both positive	‐	35,368 (1.9)	409 (64.9)	221 (35.1)	
Both negative	1,477,765	‐			1047 (100.0)
Among multi‐time participants (*n* = 728,146)					
All tests	1,143,748	298,487 (26.1)	1342 (46.5)	902 (31.2)	644 (22.3)
By sex					
Men	448,904	125,722 (28.0)	718 (46.2)	498 (32.1)	337 (21.7)
Women	694,844	172,765 (24.9)	624 (46.7)	404 (30.3)	307 (23.0)
By age group (years)					
50–59	243,732	63,600 (26.1)	205 (51.9)	125 (31.6)	65 (16.5)
60–69	673,027	174,581 (25.9)	779 (45.9)	545 (32.1)	374 (22.0)
70–74	226,989	60,306 (26.6)	358 (45.0)	232 (29.2)	205 (25.8)
By results of initial test					
RA positive only	‐	131,716 (11.5)	229 (52.8)	205 (47.2)	
FIT positive only	‐	137,315 (12.0)	903 (62.2)	549 (37.8)	
Both positive	‐	29,456 (2.6)	210 (64.0)	118 (36.0)	
Both negative	845,261	‐			644 (100.0)

Abbreviations: CRC, colorectal cancer; FIT, fecal immunochemical test; RA, risk assessment.

As shown in Table 2, parallel use of RA and 2‐specimen FIT, the initial test used in the program, achieved a sensitivity of 0.78 (0.77–0.80) and a specificity of 0.78 (0.78–0.78) in participants who joined the first round, and a sensitivity of 0.78 (0.76–0.79) and a specificity of 0.75 (0.75–0.75) in those participants who joined for more than one round. Based on one‐specimen FIT, the most commonly used initial test worldwide, the sensitivity decreased to 0.57 (0.56–0.58) among the first‐time participants and 0.49 (0.47–0.51) in the multiple‐time participants, but with elevated specificity of 0.90 or above, higher PPV, but lower detection rates of CRC when compared with parallel use of RA and 2‐specimen FIT. Regarding the other assumed initial tests, RA only and serial use of RA and FIT had a sensitivity of less than 25% in detecting CRC. However, the likelihood ratios were significant for RA only, with LR^+^ of 2.08 (1.98, 2.20) and LR^−^ of 0.87 (0.86, 0.89) for RA only. The 2‐specimen FIT alone and parallel use of RA and 1‐specimen FIT had a lower sensitivity but a higher specificity than parallel use of RA and 2‐specimen FIT.

**TABLE 2:** Diagnostic accuracy of the initial tests in the Shanghai CRC screening program 1‐year interval.

Initial tests	Positive (*n*, %)	Sensitivity (95% CI)	Specificity (95% CI)	LR^+^ (95% CI)	LR^−^ (95% CI)	Detection rate (1/1000) (95% CI)	PPV (95% CI)
Among first‐time participants (*n* = 1,901,360)							
Currently‐used test							
RA and 2‐specimen FIT (parallel)	423,595 (22.3)	0.78 (0.77, 0.80)	0.78 (0.78, 0.78)	3.54 (3.48, 3.59)	0.28 (0.26, 0.29)	1.99 (1.93, 2.05)	0.89 (0.86, 0.92)
Assumed initial test							
RA only	197,844 (10.4)	0.22 (0.20, 0.23)	0.90 (0.90, 0.90)	2.08 (1.98, 2.20)	0.87 (0.86, 0.89)	0.55 (0.52, 0.58)	0.53 (0.50, 0.56)
First FIT only	174,134 (9.2)	0.57 (0.56, 0.58)	0.91 (0.91, 0.91)	6.32 (6.16, 6.47)	0.47 (0.46, 0.49)	1.45 (1.40, 1.50)	1.58 (1.52, 1.64)
2‐specimen FIT only	261,119 (13.7)	0.70 (0.68, 0.71)	0.86 (0.86, 0.86)	5.13 (5.04, 5.23)	0.35 (0.34, 0.37)	1.77 (1.71, 1.83)	1.29 (1.25, 1.33)
RA and first FIT (parallel)	348,360 (18.3)	0.68 (0.67, 0.69)	0.82 (0.82, 0.82)	3.74 (3.66, 3.81)	0.39 (0.38, 0.41)	1.73 (1.67, 1.79)	0.94 (0.91, 0.97)
RA and first FIT (serial)	23,618 (1.2)	0.11 (0.10, 0.12)	0.99 (0.99, 0.99)	8.81 (8.11, 9.57)	0.90 (0.90, 0.91)	0.27 (0.25, 0.30)	2.19 (2.01, 2.39)
RA and 2‐specimen FIT (serial)	35,368 (1.9)	0.13 (0.12, 0.14)	0.98 (0.98, 0.98)	7.13 (6.62, 7.67)	0.89 (0.88, 0.90)	0.33 (0.31, 0.36)	1.78 (1.65, 1.92)
Among multi‐time participants (*n* = 728,146)							
Currently used method							
RA and 2‐specimen FIT (parallel)	298,487 (26.1)	0.78 (0.76, 0.79)	0.74 (0.74, 0.74)	2.99 (2.93, 3.05)	0.30 (0.28, 0.32)	1.96 (1.88, 2.04)	0.75 (0.72, 0.78)
Assumed methods							
RA only	161,172 (14.1)	0.26 (0.25, 0.28)	0.86 (0.86, 0.86)	1.88 (1.76, 2.00)	0.86 (0.84, 0.88)	0.67 (0.62, 0.72)	0.47 (0.44,0.51)
First FIT only	112,018 (9.8)	0.49 (0.47, 0.51)	0.90 (0.90, 0.90)	5.05 (4.87, 5.25)	0.56 (0.54, 0.58)	1.24 (1.17, 1.30)	1.26 (1.20,1.33)
2‐specimen FIT only	166,771 (14.6)	0.63 (0.61, 0.64)	0.86 (0.85, 0.86)	4.33 (4.21, 4.46)	0.44 (0.42, 0.46)	1.58 (1.51, 1.66)	1.09 (1.04,1.14)
RA and first FIT (parallel)	253,380 (22.2)	0.67 (0.65, 0.69)	0.78 (0.78, 0.78)	3.03 (2.96, 3.11)	0.43 (0.40, 0.45)	1.69 (1.61, 1.77)	0.76 (0.73,0.80)
RA and first FIT (serial)	19,810 (1.7)	0.09 (0.07, 0.10)	0.98 (0.98, 0.98)	4.97 (4.40, 5.60)	0.93 (0.92, 0.94)	0.22 (0.19, 0.24)	1.24 (1.09,1.41)
RA and 2‐specimen FIT (serial)	29,456 (2.6)	0.11 (0.10, 0.13)	0.97 (0.97, 0.97)	4.45 (4.01, 4.93)	0.91 (0.90, 0.92)	0.29 (0.26, 0.32)	1.11 (1.00,1.24)

*Note*: 2‐sample FIT‐positive referring to positive in any FIT.

Abbreviations: CI, confidence interval; FIT, fecal immunochemical test; LR^+^, positive likelihood ratio; LR^−^, negative likelihood ratio; PPV, positive predict value; RA, risk assessment.

### 3.2 Meta‐analysis

**FIGURE 2:**
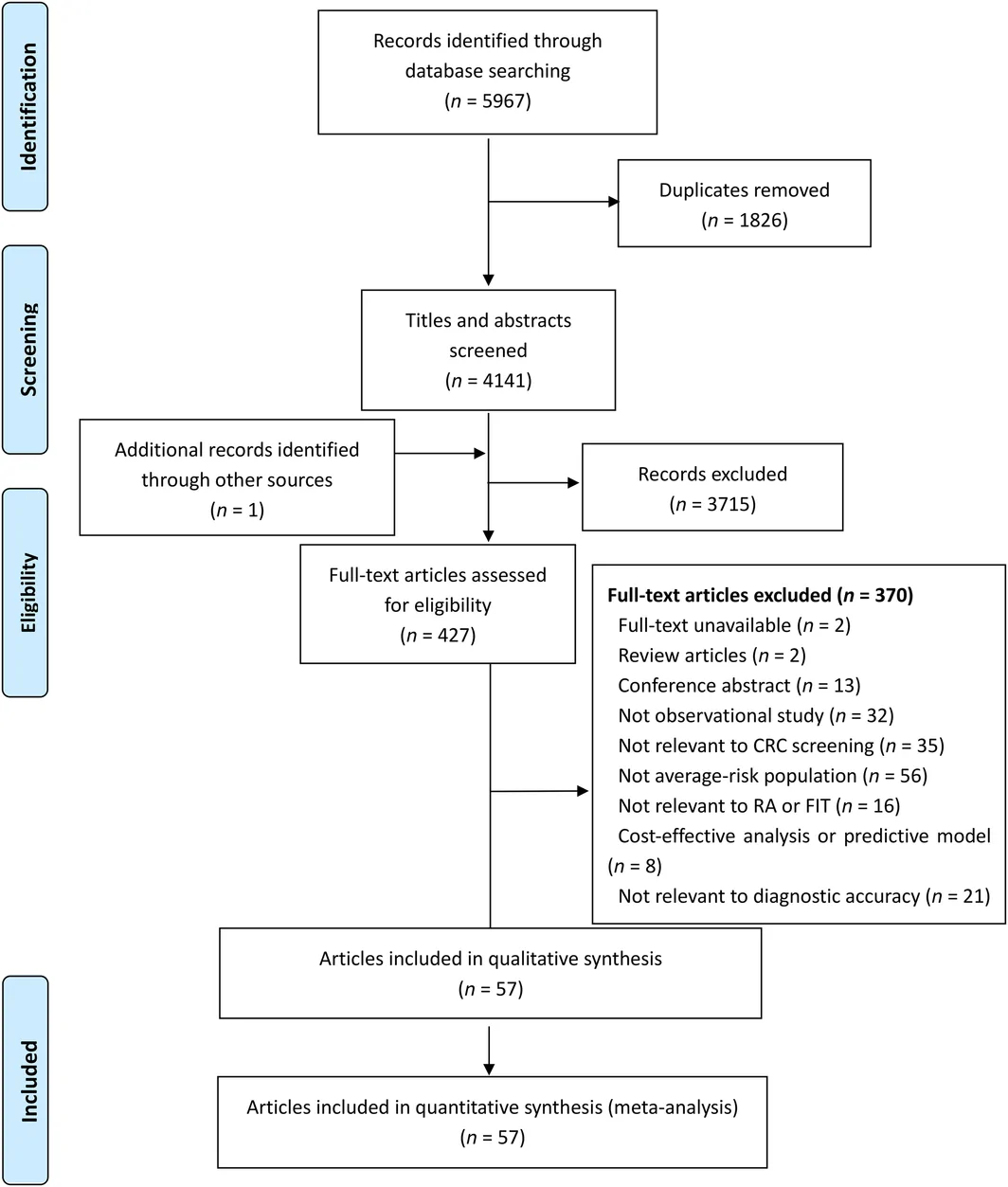
Literature search and selection of studies for meta‐analysis.

The details and the results of literature search and selection of studies for meta‐analysis are presented in Figure 2. The characteristics of the 103 eligible studies from 57 selected articles published during 1992–2021 are shown in Table S2. A total of 25,111,195 participants from Asian, European, and American countries were involved. Of the 103 studies, 92 used FIT only as the initial test (*n* = 23,198,276), 5 performed RA only (*n* = 688,579), 3 used RA and FIT parallel (*n* = 147,042), and 3 applied RA and FIT serial (*n* = 1,077,298). Among the 93 studies using FIT alone, 66 used 1 specimen and 12 applied 2 specimens, whereas 66 engaged quantitative methods and 25 used qualitative methods, with a threshold for a positive result ranging from 4 to 300 μg Hb/g faces. Figure S1 and Table S3 demonstrate the details of quality assessment for the included studies, showing the greatest risk of bias in “patient selection.” The Deek's funnel plot asymmetry test suggested low risk of publication bias in all included studies (*p* = 0.57) (Figure S2).

The pooled sensitivity, specificity, PPV, and detection rate of CRC for all published studies were 0.73 (0.69–0.77), 0.92 (0.90–0.94), 4.80% (4.37, 5.23), 2.87 (2.61–3.15) per 1000, respectively (Table 3). The summary performance reported in these previous studies were better than the Shanghai program, particularly for 1‐specimen FIT. Interestingly, the pooled specificity of parallel use of 2‐specimen FIT and RA was 0.42 (0.13, 0.79) in previous studies, much lower than those in the first‐time participants (0.78) and the multiple‐time participants (0.74) of the Shanghai program. The pooled LR^+^ and LR^−^ for RA only were 1.77 (0.77, 4.01) and 0.83 (0.58, 1.24), respectively, as shown in Table S4.

**TABLE 3:** Summarized accuracy of initial tests for CRC screening in previous observational studies.

	No. of studies	Pooled sensitivity (95% CI)	*I* ^2^	Pooled specificity (95% CI)	*I* ^2^	Detection rate (1/1000) (95% CI)	*I* ^2^	Pooled PPV (%) (95% CI)	*I* ^2^
Overall	103	0.73 (0.69, 0.77)	99.2	0.92 (0.90, 0.94)	100.0	2.87 (2.61, 3.15)	99.4	4.80 (4.37, 5.23)	99.7
Population									
Chinese	28	0.56 (0.46, 0.66)	99.7	0.91 (0.85, 0.94)	100.0	1.67 (1.25, 2.09)	99.7	3.23 (2.63, 3.83)	99.8
Other Asian populations	40	0.61 (0.53, 0.68)	99.5	0.91 (0.88, 0.94)	100.0	2.16 (1.78, 2.53)	99.6	3.88 (3.30, 4.47)	99.8
Western populations	63	0.81 (0.77, 0.84)	89.2	0.93 (0.92, 0.94)	99.8	3.35 (2.94, 3.77)	98.6	5.20 (4.69, 5.72)	97.1
Type of screening									
Organized	67	0.74 (0.68, 0.79)	99.2	0.94 (0.92, 0.95)	100.0	2.46 (2.18, 2.75)	99.6	4.23 (3.74, 4.72)	99.8
Opportunistic	36	0.71 (0.66, 0.75)	59.6	0.89 (0.85, 0.92)	99.4	6.88 (5.55, 8.20)	84.4	7.94 (6.37, 9.51)	90.7
Wave of screening									
First	48	0.72 (0.65, 0.79)	99.1	0.94 (0.92, 0.95)	100.0	2.35 (2.04, 2.66)	99.6	4.25 (3.71, 4.78)	99.8
Subsequent	19	0.77 (0.69, 0.84)	98.3	0.93 (0.90, 0.95)	99.9	2.74 (2.12, 3.36)	99.5	4.08 (3.36, 4.80)	98.9
Type of initial test									
FIT only	92	0.76 (0.72, 0.79)	98.7	0.93 (0.92, 0.95)	99.9	3.01 (2.74, 3.28)	99.1	5.26 (4.81, 5.71)	99.3
RA only	5	0.38 (0.20, 0.60)	96.2	0.72 (0.54, 0.95)	100.0	0.81 (0.44, 1.17)	82.2	0.71 (0.21, 1.21)	93.8
RA and FIT (parallel)	3	0.76 (0.36, 0.94)	84.3	0.59 (0.28, 0.85)	99.9	2.29 (1.99, 2.58)	99.3	0.86 (0.36, 1.35)	76.4
RA and FIT (serial)	3	0.24 (0.04, 0.69)	90.3	0.97 (0.93, 0.99)	100.0	0.28 (0.18, 0.38)	81.5	1.53 (0.00, 3.13)	98.1
No. of specimen for FIT									
1‐specimen	66	0.76 (0.72, 0.80)	98.3	0.92 (0.90, 0.94)	99.9	3.73 (3.34, 4.12)	99.0	6.08 (5.45, 6.72)	99.5
2‐specimen	12	0.76 (0.61, 0.87)	98.9	0.94 (0.92, 0.96)	99.9	2.11 (1.46, 2.76)	97.5	3.45 (2.72, 4.18)	92.2
Type of FIT									
Quantitative	66	0.77 (0.73, 0.80)	95.5	0.92 (0.90, 0.94)	99.9	3.92 (3.51, 4.32)	98.1	5.76 (5.34, 6.18)	94.5
Qualitative	25	0.73 (0.66, 0.80)	99.3	0.95 (0.94, 0.96)	100.0	1.93 (1.61, 2.24)	99.3	3.68 (3.05, 4.31)	99.6
Cutoff for FIT (μg/g)^a^									
<20	26	0.83 (0.78, 0.86)	63.7	0.89 (0.86, 0.91)	99.7	5.91 (4.72, 7.11)	95.7	5.28 (4.52, 6.04)	88.8
20	32	0.76 (0.70, 0.82)	99.4	0.94 (0.93, 0.95)	100.0	2.30 (1.92, 2.67)	99.5	4.44 (3.74, 5.13)	99.7
>20	28	0.72 (0.66, 0.78)	88.2	0.94 (0.91, 0.96)	99.7	3.50 (2.70, 4.30)	96.8	7.16 (6.30, 8.03)	83.8

Abbreviations: CI, confidence interval; FIT, fecal immunochemical test; PPV, positive predict value; RA, risk assessment.

^a^ Cutoff value under 20 μg/g ranging from 4 to 17 μg/g; cutoff value over 20 μg/g ranging from 25 to 300 μg/g.

**FIGURE 3:**
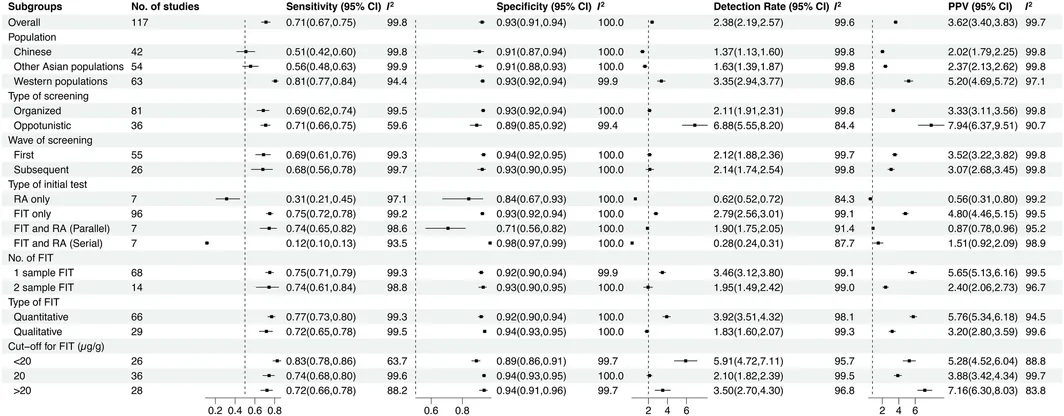
Summarized sensitivity, specificity, PPV, and detection rate of initial tests for CRC screening. CI, confidence interval; CRC, colorectal cancer; FIT, fecal immunochemical test; PPV, positive predictive value; RA, risk assessment.

Including our results in meta‐analysis leads to slight changes in the summary estimates, with a lower pooled sensitivity [0.71 (0.67–0.75)], a higher specificity [0.93 (0.91–0.94)], a lower detection rate [2.38 (2.19–2.57) per 1000], and a lower PPV [3.62% (3.40–3.83)] (Figure 3). Regardless of the number of FIT, the changes were moderate for FIT only, but substantially for parallel use of RA and FIT as the initial test. Stratified meta‐analysis demonstrated that type of screening (opportunistic/organized) and wave of screening (first/subsequent) contributed to the heterogeneities in performance of initial tests, while the population (Chinese/other Asian populations/western populations), number of FIT (1‐/2‐specimen), type of FIT (qualitative/quantitative), and threshold for positive FIT (<20/20/>20 μg/g) were not significant contributors. Further meta‐regression analyses demonstrated that the sensitivity of initial tests were highly heterogeneous with respect to population, type of initial test [RA only, FIT only, RA and FIT (parallel), RA and FIT (serial)], type of FIT, and threshold for positive FIT. The type of initial test explained 82.90% of total between‐study heterogeneity in sensitivity (*p* < 0.01). Regarding the specificity, the population, year of screening (before/after 2005), sample size (</≥10,000), type of initial test, and threshold for positive FIT were significant in multivariable meta‐regression model, with the type of initial test accounting for 69.55% of total between‐study heterogeneity in specificity (*p* < 0.01), as shown in Table S5.

Sensitivity analysis by excluding studies with high risk of bias or participants less than 1000 did not observe substantial changes in pooled sensitivity, specificity, AUC, and detection rate of CRC (Figure S3).

As shown in Fagan's plot (Figure S4), the posttest probability for a negative result was 0.0027 for FIT only and 0.0041 for parallel test of FIT and RA, but were 0.10 and 0.02 for a positive result, respectively. Based on these findings, we estimated that 11 more CRC cases could be detected from 100,000 screened subjects using parallel test of FIT and RA than FIT alone, but at the cost of additional 33,504 false‐positive results.

We further compared the characteristics and results of the current meta‐analysis with previous publications. As shown in Table S6, the pooled sensitivity, specificity, LR^+^, and LR^−^ were close to those summarized in Lee et al.'s and Stonestreet et al.'s analyses.

## 4 DISCUSSION

This study evaluated the diagnostic accuracy of RA and FIT, both being used alone and combined for CRC screening, in average‐risk populations. In the large‐scale population‐based screening program in Shanghai, China, we observed acceptable sensitivity and specificity for parallel use of RA and FIT, but there still exists a large number of missed cases due to poor compliance to subsequent colonoscopy. In the meta‐analysis, we found that FIT was used alone as an initial test in 93 of 103 studies, achieving a pooled sensitivity of 76% and specificity of 93%; only four studies used RA and FIT in parallel, with a higher summary sensitivity (83%) but a much lower pooled specificity (42%) than FIT alone. It is evident that Chinese populations can benefit more from parallel use of RA and FIT for initial screening of CRC than for other populations, probably due to the higher proportion of non‐bleeding colorectal lesions in the population.^9^ Our results highlighted the value of FIT in CRC screening as an initial test, and demonstrated that RA can be used with FIT in parallel in Chinese populations—conditional upon good compliance to subsequent colonoscopy.

FIT is a dominant initial test in triage screening of CRC. Our results derived from the Shanghai CRC screening program and a meta‐analysis of population‐based screening programs revealed the diagnostic value of the test in any rounds of organized CRC screening programs. In meta‐analysis, we did not find significant differences in sensitivity and specificity of FIT by the numbers of stool specimens tested or the type of FIT, which is consistent with a previous systematic review^26^; however, we observed a higher detection rate and PPV for 1‐specimen FIT, which may be explained by the quantitative method mainly used for 1‐specimen test in previous studies. Regarding the positivity threshold that ranged from 4 to 300 μg/g for FIT worldwide, we found that the cutoff points were more likely to have an impact on sensitivity and LR^−^ than on specificity and LR^+^, which were in line with a previous systematic review.^27^ The threshold lower than 20 μg/g had the highest sensitivity (0.82), but the lowest specificity (0.89) and the lowest LR^−^ (0.20). However, one could not determine the optimal threshold based on diagnostic accuracy only, but also should take the cost‐effectiveness into consideration. Evidently, a trade‐off should be made according to the availability of health resources. In fact, qualitative FIT, usually with a cutoff point of 20 μg/g, is still widely used in CRC screening due to its convenience and low cost.

Multiple RA systems have been developed based on risk factors identified in different populations. The RA system used in this study was the recommended one based on specified factors in Chinese populations such as intestinal symptoms, psychological status, chronic cholecystitis, cholecystectomy, and family history of CRC.^9^ The RA system demonstrated a sensitivity of 0.25 and a specificity of 0.90 in a Chinese population aged 40–74 years,^28^ and a sensitivity of 0.57 and a specificity of 0.91 in our subjects. It seems that RA had a much higher specificity in Chinese populations than in other population. Moreover, as LR^+^ and LR^−^ were significant for RA only in our populations, indicating the value of the test in Chinese populations.

RA is rarely used alone due to its low sensitivity, but usually applied in parallel with FIT for its superior performance in detecting non‐bleeding colorectal lesions.^9^, ^29^ FIT, on the other hand, presented a lower accuracy in Asians, partly due to the higher proportion of non‐bleeding lesions in the populations.^30^ It is of note that, parallel use of RA and FIT had a slightly higher sensitivity than FIT alone (0.78 vs. 0.75) in our subjects, but with a compromise on specificity (0.56 vs. 0.94), leading to more missed cases (2743 of 5493 positive cases, 49.9%) than using FIT alone (2258 of 4753 positive cases, 47.5%). The results can be explained by the lower adherence to colonoscopy among subjects with positive results in RA or FIT than those positive in FIT, as we described previously.^31^ It seems that the false positive results raised as the true positive results increased, leading to distrust of the initial test, and thus a low adherence to colonoscopy. Obviously, attempt should be made to improve adherence to colonoscopy to achieve the maximal effectiveness of the screening.

The strengths of the study included the large sample size of the Shanghai CRC screening program, the available test results of RA and 2‐specimen FIT for all participants, and the supplemented information on prevalent CRC by a record linkage with the Shanghai cancer registry. These led to accurate estimations of the performance of multiple initial screening methods. Moreover, our meta‐analysis followed the recommendations of PRISMA on retrieval of databases with robust data extraction and quality assessment. It included observational studies only and represented the real‐world population‐based CRC screening worldwide. Furthermore, the large number of studies included in meta‐analysis enabled us to comprehensively evaluate the diagnostic accuracy of multiple initial tests.

However, there existed significant heterogeneity in diagnostic accuracy of initial tests across subgroups in meta‐analysis, which may be attributed to different demographic characteristics of screened populations, varied prevalence of CRC, stage of CRC, location of colorectal lesions, and other influencing factors.^32^, ^33^, ^34^ In this study, however, we found that population explained only 21.63% heterogeneity in sensitivity and less than 0.01% heterogeneity in specificity. The type of initial test was the most contributor to the heterogeneities in diagnostic accuracy. Furthermore, CRC screening is usually recommended for people over 50 years.^35^ However, several studies included in meta‐analysis enrolled people younger than 50 years old, and the findings should be interpreted with caution. Finally, there were only 13 studies using RA alone or combined with FIT as an initial test, restricting more detailed analyses and comparisons of diagnostic accuracy of different RA systems in CRC screening.

## 5 CONCLUSIONS

In conclusion, our results derived from the Shanghai CRC screening program and a meta‐analysis of population‐based screening programs support the utility of FIT in initial screening of CRC. Parallel use of RA with FIT may help to detect the higher proportion of non‐bleeding lesions in Chinese populations and improve the sensitivity of initial test, but may decrease the specificity. Our results suggest that the RA system should be optimized to guarantee the effectiveness and cost‐effectiveness of the triage screening of CRC in Chinese populations.

## AUTHOR CONTRIBUTIONS

**Ziyang Wang:** Formal analysis (equal); writing – original draft (equal). **Jiaoyue Teng:** Formal analysis (equal). **Weimiao Wu:** Formal analysis (equal). **Jianming Dou:** Data curation (equal). **Martin C. S. Wong:** Writing – review and editing (equal). **Yangming Gong:** Data curation (equal). **Junjie Huang:** Writing – review and editing (equal). **Kai Gu:** Conceptualization (equal); project administration (equal); writing – review and editing (equal). **Wanghong Xu:** Conceptualization (lead); project administration (equal); writing – review and editing (equal).

## FUNDING INFORMATION

This study was supported by the Health Commission of the Pudong New Area of Shanghai (No. PW2019A‐5) and the Key Technology Research for Colorectal Cancer Screening and High‐risk Population Follow‐up (No. 20DZ1100103).

## CONFLICT OF INTEREST STATEMENT

All authors declare no competing interests.

## ETHICS STATEMENT

The Shanghai CRC screening program was approved by the Ethical Review Committee of the Shanghai Municipal Center for Disease Control & Prevention (approval number: 2020‐31).

## CONSENT FOR PUBLICATION

The study participants provided written consent for the publication of this information.

## Supporting information


Data S1.

## Data Availability

The datasets generated and/or analyzed during the current study are available from the corresponding author on reasonable request.
