# Supplementary material for: Diagnostic accuracy of risk assessment and fecal immunochemical test in colorectal cancer screening: Results from a population‐based program and meta‐analysis
Source: Cancer Med. 2023 Aug 14;12(17):18189–200. doi: 10.1002/cam4.6399 (PMC10524075; doi:10.1002/cam4.6399)
Supplement: Supplementary file 1 — Data S1. [file CAM4-12-18189-s001.pdf]

Table S1 Strategy for literature searching

Databases	Search strategy
Pubmed	(colorect*[TIAB] OR colon*[TIAB] OR rectal[TIAB] OR rectum[TIAB] OR intestin*[TIAB] OR bowel[TIAB] OR Intestine, Large[MeSH])<br>AND (cancer*[TIAB] OR neoplas*[TIAB] OR carcinom*[TIAB] OR tumor[TIAB] OR tumors[TIAB] OR tumour* OR "Neoplasms"[MeSH])<br>AND ("Early Diagnosis"[MeSH] OR "Mass Screening"[MeSH] OR screen[TIAB] OR screening[TIAB] OR surveillance[TIAB] OR early diagnosis[TIAB] OR diagnostic test[TIAB] OR diagnostic tests[TIAB] OR early detection[TIAB])<br>AND (risk assessment[TIAB] OR risk stratification[TIAB] OR risk score[TIAB] OR risk evaluation[TIAB] OR risk classification[TIAB] OR questionnaire[TIAB] OR HRFQ[TIAB] OR RA[TIAB] OR FOBT[TIAB] OR FIT[TIAB] OR iFOBT[TIAB] OR ((faecal[TIAB] OR fecal[TIAB] OR feces[TIAB] OR faeces[TIAB] OR stool*[TIAB]) AND ("Occult Blood"[MeSH] OR occult blood[TIAB] OR immunochem* [TIAB] OR immunohistochem*[TIAB])))<br>AND (sensitivity OR specificity OR accuracy OR “Sensitivity and Specificity”[MeSH] OR “predictive value” OR “Predictive Value of Tests”[MeSH])<br>Filters=: Publication date to 2022/1/5, Humans, English.
Embase	#1 exp large intestine/<br>#2 (colorect* or colon* or rectum or rectal or intestin* or bowel).ab,kw,ti.<br>#3 1 or 2<br>#4 exp neoplasm/<br>#5 (cancer* or neoplas* or carcinom* or tumor* or tumour*).ab,kw,ti.<br>#6 4 or 5<br>#7 exp screening/ or exp early diagnosis/ or exp mass screening/ or early cancer diagnosis/<br>#8 (screen or screening or surveillance or early diagnosis or diagnostic test or diagnostic tests or early detection).ab,kw,ti.<br>#9 7 or 8<br>#10 (risk assessment or risk stratification or risk score or risk evaluation or risk classification or questionnaire or HRFQ or RA or FOBT or FIT or iFOBT).ab,kw,ti.<br>#11 (faecal or fecal or feces or faeces or stool*).ab,kw,ti.<br>#12 exp occult blood/<br>#13 (occult blood or immunochem* or immunohistochem*) .ti,ab,kw.<br>#14 12 or 13<br>#15 11 and 14<br>#16 10 or 15<br>#17 exp "sensitivity and specificity"/ or exp predictive value/ or exp diagnostic accuracy/ or diagnostic value/ or diagnostic test accuracy study/<br>#18 (accuracy or sensitivity or specificity or predictive value).ab,kw,ti.<br>#19 17 or 18<br>#20 3 and 6 and 9 and 16 and 19<br>#21 limit 20 to (human and english language and yr="1883 - 2022")

Web of science	TS= (colorect* OR colon* OR rectal OR rectum OR intestin*OR bowel) AND TS= (cancer*OR neoplas* OR carcinom* OR tumor* OR tumour*) AND TS= (screen OR screening OR surveillance OR "early diagnosis" OR "diagnostic test" OR "diagnostic tests" OR "early detection") AND TS=("risk assessment" OR "risk stratification" OR "risk score" OR "risk evaluation" OR "risk classification" OR questionnaire OR HRFQ OR RA OR FOBT OR FIT OR iFOBT OR ((faecal OR fecal OR feces OR faeces OR stool*) AND ("occult blood" OR immunochem* OR immunohistochem*))) AND TS=(accuracy OR sensitivity OR specificity OR "predictive value")
Cochrane Library	#1 MeSH descriptor: [Intestine, Large] explode all trees<br>#2 (colorect* OR colon* OR rectum OR rectal OR intestin* OR bowel):ti,ab,kw<br>#3 #1 OR #2<br>#4 MeSH descriptor: [Neoplasms] explode all trees<br>#5 (cancer* OR neoplas* OR carcinom* OR tumor* OR tumour*):ti,ab,kw<br>#6 #4 OR #5<br>#7 MeSH descriptor: [Early Diagnosis] explode all trees<br>#8 MeSH descriptor: [Mass Screening] explode all trees<br>#9 (screen OR screening OR surveillance OR "early diagnosis" OR "diagnostic test" OR "diagnostic tests" OR "early detection"):ti,ab,kw<br>#10 #7 OR #8 OR #9<br>#11 ("risk assessment" OR "risk stratification" OR "risk score" OR "risk evaluation" OR "risk classification" OR questionnaire OR HRFQ OR RA OR FOBT OR FIT OR iFOBT):ti,ab,kw<br>#12 (faecal OR fecal OR feces OR faeces OR stool*):ti,ab,kw<br>#13 MeSH descriptor: [Occult Blood] explode all trees<br>#14 ("occult blood" OR immunohistochem* OR immunochem*):ti,ab,kw<br>#15 #12 AND (#13 OR #14)<br>#16 #11 OR #15<br>#17 MeSH descriptor: [Sensitivity and Specificity] explode all trees<br>#18 (accuracy OR sensitivity OR specificity OR “predictive value”)<br>#19 #17 OR #18<br>#20 #3 AND #6 AND #10 AND #16 AND #19

Table S2 Characteristics of the studies included in current systematic review and meta-analysis

First author	Publication Year	Country/Area	Initial test	Recruitment	Wave of screening	FIT			Test results			
						Type	No. of fecal sample	Cut-off value (μg/g)	TP	FP	FN	TN
Wu	2019	China	FIT	Organized	First	Qualitative	1	20	427	69846	796	467209
Wu	2019	China	FIT	Organized	First	Qualitative	2	20	236	14052	987	523003
Wu	2019	China	RA	Organized	First				125	28668	1098	508387
Wu	2019	China	RA & FIT (serial)	Organized	First	Qualitative	1	20	172	8100	1051	528955
Wu	2019	China	RA & FIT (serial)	Organized	First	Qualitative	2	20	312	247502	911	289553
Chen	2018	China	FIT	Organized	First	Qualitative	1	20	1496	26894	509	694214
Cubiella	2014	Spain	FIT	Opportunistic		Quantitative	1	20	3	47	0	672
Wu	2014	China	FIT	Opportunistic		Qualitative	1	10	3	35	2	967
Kim	2016	Korea	FIT	Organized	First	Quantitative	1	20	58	681	21	3230
Chen	2016	China	FIT	Organized	First	Qualitative	1	20	859	20494	62	490651
Brenner	2017	Germany	FIT	Opportunistic		Quantitative	1	17	29	346	1	3118
Yuan	2019	China	FIT	Opportunistic		Quantitative	1	30	29	531	12	120
Yuan	2019	China	FIT	Opportunistic		Quantitative	1	40	28	279	13	372
Yuan	2019	China	FIT	Opportunistic		Quantitative	1	60	26	192	15	459
Yuan	2019	China	FIT	Opportunistic		Quantitative	1	80	25	99	16	552
Yuan	2019	China	FIT	Opportunistic		Quantitative	1	100	25	71	16	580
Yuan	2019	China	FIT	Opportunistic		Quantitative	1	120	22	56	19	595
Kim	2017	Korea	FIT	Organized	First	Quantitative	1	20	59	679	21	3231
Allison	2007	USA	FIT	Organized	First	Qualitative	1	300	9	164	2	5181
Brenner	2013	Germany	FIT	Opportunistic		Quantitative	1	6.1	11	99	4	2121
Shin	2013	Korea	FIT	Organized	Subsequent	Qualitative	1		374	23755	270	329615

Shin	2013	Korea	FIT	Organized	First	Quantitative	1	20	2049	120108	1268	1326376
Shin	2013	Korea	FIT	Organized	First	Qualitative	1		434	9497	405	283208
Shin	2013	Korea	FIT	Organized	Subsequent	Quantitative	1	20	95	2054	97	87094
Brenner	2018	Germany	FIT	Opportunistic		Quantitative	1	10	24	487	1	1799
Brenner	2018	Germany	FIT	Opportunistic		Quantitative	1	17	23	292	2	2894
Brenner	2018	Germany	FIT	Opportunistic		Quantitative	1	20	23	247	2	2939
Brenner	2018	Germany	FIT	Opportunistic		Quantitative	1	30	22	180	3	3006
Brenner	2018	Germany	FIT	Opportunistic		Quantitative	1	40	20	147	5	3039
Brenner	2018	Germany	FIT	Opportunistic		Quantitative	1	50	18	126	7	3060
Chen	2014	China	FIT	Opportunistic		Qualitative		10	9	220	4	5863
Hijos-Mallada	2021	Spain	FIT	Organized	First	Qualitative	1	5	19	126	3	188
Hijos-Mallada	2021	Spain	FIT	Organized	First	Qualitative	1	20	16	57	6	257
Ye	2017	China	FIT & RA (parallel)	Organized	First	Qualitative	2	20	219	17845	34	77945
Ye	2017	China	FIT	Organized	First	Qualitative	2	20	191	9237	62	86553
Ye	2017	China	RA	Organized	First				62	9788	191	86002
EDWARDS	2004	Italy	FIT	Opportunistic		Qualitative	1	200	147	1833	49	33110
Castiglione	1996	Italy	FIT	Organized	First	Qualitative	1		15	468	7	7518
He	2019	China	FIT & RA (Serial)	Opportunistic		Qualitative	1	40	4	48	1	1148
He	2019	China	FIT & RA (parallel)	Opportunistic		Qualitative	1	40	5	846	0	350
Aniwan	2016	Thailand	FIT	Opportunistic		Quantitative	1	5	11	260	3	1205
Aniwan	2016	Thailand	FIT	Opportunistic		Quantitative	1	10	11	165	3	1300
Aniwan	2016	Thailand	FIT	Opportunistic		Quantitative	1	20	11	97	3	1368
Aniwan	2016	Thailand	FIT	Opportunistic		Quantitative	1	30	11	76	3	1389
Aniwan	2016	Thailand	FIT	Opportunistic		Quantitative	1	40	9	63	5	1402
Rubeca	2012	Italy	FIT	Organized	First	Quantitative	2	40	9	261	0	4718
Rubeca	2012	Italy	FIT	Organized	First	Quantitative	2	40	9	222	0	4757

Rubeca	2012	Italy	FIT	Organized	First	Quantitative	2	40	6	139	3	4840
Raginel	2013	France	FIT	Organized	First	Quantitative	1	180	28	362	19	19388
Raginel	2013	France	FIT	Organized	First	Quantitative	2	180	36	596	11	19154
Raginel	2013	France	FIT	Organized	First	Quantitative	1	80	37	511	10	19239
Raginel	2013	France	FIT	Organized	First	Quantitative	1	30	37	514	10	19236
Raginel	2013	France	FIT	Organized	First	Quantitative	2	30	44	757	3	18992
Raginel	2013	France	FIT	Organized	First	Quantitative	1	20	44	650	3	19100
Nakama	1996	Japan	FIT	Organized	First	Qualitative			10	147	2	3206
Nakama	2001	Japan	FIT	Organized	First	Qualitative	1		5	97	2	1584
Masuda	2011	Japan	FIT	Opportunistic		Quantitative	1	5	6	294	2	783
Masuda	2011	Japan	FIT	Opportunistic		Quantitative	1	10	6	144	2	933
Masuda	2011	Japan	FIT	Opportunistic		Quantitative	1	15	4	96	4	981
Masuda	2011	Japan	FIT	Opportunistic		Quantitative	1	20	4	74	4	1003
Masuda	2011	Japan	FIT	Opportunistic		Quantitative	1	25	2	55	6	1022
Masuda	2011	Japan	FIT	Opportunistic		Quantitative	1	30	2	48	6	1029
Wong	2015	China	FIT	Organized	First	Qualitative	2	10	12	510	10	4811
Jung	2020	Korea	FIT	Organized	First	Qualitative		20	9912	309824	5551	5093973
Vanaclocha-Espi	2021	Spain	FIT	Organized	First	Qualitative	1	20	1969	34925	216	508395
Chiang	2014	China	FIT	Organized	First	Quantitative	1	20	359	7762	128	200680
Chiang	2014	China	FIT	Organized	First	Quantitative	1	20	1546	26560	460	718510
Itoh	1996	Japan	FIT	Organized	First	Qualitative		10	77	1413	12	26358
Launoy	2005	France	FIT	Organized	First	Qualitative	2	4	24	410	4	6983
Jensen	2016	USA	FIT	Organized	First	Qualitative		20	545	15492	100	307212
Jensen	2016	USA	FIT	Organized	Subsequent	Qualitative		20	147	6996	48	176797
Jensen	2016	USA	FIT	Organized	Subsequent	Qualitative		20	138	5964	50	157129
Jensen	2016	USA	FIT	Organized	Subsequent	Qualitative		20	128	6083	36	139205

Zorzi	2011	Italy	FIT	Organized	First	Quantitative	1	20	202	4502	39	82366
Zorzi	2011	Italy	FIT	Organized	First	Qualitative	1	20	147	3061	29	62232
Mlakar	2018	Slovenia	FIT	Organized	Subsequent	Quantitative	2	20	493	14654	79	273844
Digby	2016	The UK	FIT	Organized	Subsequent	Quantitative	1	80	30	723	31	30109
Parente	2013	Italy	FIT	Organized	First	Quantitative	1	20	95	2311	8	36393
Chou	2016	China	FIT	Organized	Subsequent	Quantitative	1	20	7479	167151	930	2027441
Arana-Arri	2017	Spain	FIT	Organized	First	Qualitative		20	1032	17241	136	277969
Chiu	2016	Multiple	RA	Organized	First				25	1751	16	4418
Levi	2007	Israel	RA	Opportunistic					3	102	0	115
Castiglione	2007	Italy	FIT	Organized	Subsequent	Quantitative		20	67	1030	16	26390
Allison	1996	USA	FIT	Opportunistic		Quantitative	NA	200	22	418	10	7043
Chen	2011	China	FIT	Organized	Subsequent	Quantitative	1	20	91	1940	111	44213
Levi	2011	Israel	FIT	Organized	Subsequent	Quantitative	3	14	6	147	0	1071
Mansouri	2016	UK	FIT	Organized	Subsequent			50	479	5680	182	198194
Niv	1992	Israel	FIT & RA (parallel)	Organized	Subsequent				4	665	6	1122
Niv	1992	Israel	RA	Organized	First				2	27	4	1091
Parra-Blanco	2010	Spain	FIT	Organized	First	Quantitative	1	10	14	160	0	1582
Teixeira	2017	Brazil	FIT	Opportunistic		Quantitative	1	10	11	81	1	855
Bretagne	2019	France	FIT	Organized	Subsequent	Quantitative	1	30	370	4711	25	114117
Guo	2020	Belgium	FIT	Organized	Subsequent	Quantitative	1	15	164	2021	41	25991
Schreuders	2019	Netherlands	FIT	Organized	Subsequent	Quantitative	1	10	53	1354	8	5895
Schreuders	2019	Netherlands	FIT	Organized	Subsequent	Quantitative	2	10	26	619	2	2020
van de Veerdonk	2020	Belgium	FIT	Organized	Subsequent	Quantitative	1	15	4094	84781	497	1122982
Toes-Zoutendijk	2020	Netherlands	FIT	Organized	First	Quantitative	1	15	1102	14509	126	111674
Toes-Zoutendijk	2020	Netherlands	FIT	Organized	First	Quantitative	1	47	2108	23223	418	372756

Njor	2021	Denmark	FIT	Organized	First	Quantitative	1	20	2086	33496	461	495785
Van der Vlugt	2017	Netherlands	FIT	Organized	Subsequent	Quantitative	1	10	133	2872	27	15684
Mattar	2020	Brazil	FIT	Opportunistic		Quantitative	2	10	6	17	2	92
Mattar	2020	Brazil	FIT	Opportunistic		Quantitative	1	10	5	22	1	144
Nakazato	2006	Japan	FIT	Organized	First	Quantitative			15	585	13	7184

Abbreviations: RA: risk assessment; FIT: fecal immunochemical test; TP: True positive; FP: False positive; FN: False negative; TN: True negative.

Table S3. Summarized risk of bias and applicability concerns: authors' assessment for each domain in each study included

Study	RISK OF BIAS				APPLICABILITY CONCERNS		
	PATIENT SELECTION	INDEX TEST	REFERENCE STANDARD	FLOW AND TIMING	PATIENT SELECTION	INDEX TEST	REFERENCE STANDARD
Study 1	☺	☺	?	☺	☺	☺	☺
Study 2	☺	☺	?	☹	☺	☺	☺
Study 3	☺	☺	☺	☺	☺	☺	☺
Study 4	☺	☺	?	☺	☺	☺	☺
Study 5	☺	☺	?	☺	☺	☺	☺
Study 6	?	☺	?	?	?	?	?
Study 7	☺	☺	☺	☺	☺	☺	☺
Study 8	☺	☺	☺	☺	☺	☺	☺
Study 9	☺	☺	?	☺	☺	☺	☺
Study 10	☺	☺	?	☺	☺	☺	☺
Study 11	☺	☺	☺	☺	☺	☺	☺
Study 12	?	☺	?	☺	☺	☺	☺
Study 13	☺	☺	☺	☺	☺	☺	☺
Study 14	☺	☺	☺	☺	☺	☺	☺
Study 15	☺	☺	?	☺	☺	☺	☺
Study 16	☺	?	?	☹	☺	☺	☺
Study 17	?	☺	?	?	?	?	☺
Study 18	☹	☺	?	☹	?	☹	☺
Study 19	☺	☺	☺	☺	☺	☺	☺
Study 20	?	☺	☺	☺	☺	☺	☺
Study 21	☺	☺	☺	☺	☺	☺	☺
Study 22	☺	☺	☹	☺	☺	☺	☺
Study 23	☺	?	☺	☺	?	☺	☺
Study 24	☺	?	?	☺	☺	☺	☺
Study 25	☺	?	☺	?	☺	☺	☺
Study 26	☺	?	?	?	?	☺	☺
Study 27	☺	?	?	☺	☺	☺	☺
Study 28	☺	?	☺	?	☺	☺	☺
Study 29	☺	☺	?	☺	☺	☺	☺
Study 30	?	☺	☹	☺	☺	☺	☺
Study 31	?	☺	?	☺	☺	☺	☺
Study 32	?	☺	?	☺	☺	☺	☺
Study 33	☺	☺	?	☺	☺	☺	☺
Study 34	☺	☺	?	☺	☺	☺	☺
Study 35	☺	☺	?	☺	☺	☺	☺

Study 36	☺	☺	?	☺	☺	☺	☺
Study 37	☺	☺	?	☺	☺	☺	☺
Study 38	☺	☺	?	☺	☺	☺	☺
Study 39	☺	☺	☺	☺	☺	☺	☺
Study 40	☺	☺	?	☺	☺	☺	☺
Study 41	?	☺	?	☺	☺	☺	☺
Study 42	☺	☺	?	☺	☺	☺	☺
Study 43	☺	☺	?	☺	☹	☺	☺
Study 44	☺	☺	?	☺	☺	☺	☺
Study 45	☺	☺	?	☺	☺	☺	☺
Study 46	☺	☺	?	☺	☹	☺	☺
Study 47	☺	☺	?	☺	☺	☺	☺
Study 48	☺	☺	?	☺	☺	☺	☺
Study 49	☺	☺	☺	☺	☺	☺	☺
Study 50	☺	☺	?	☺	☺	☺	☺
Study 51	☺	☹	?	☺	☺	☺	☺
Study 52	☺	☺	?	☺	☺	☺	☺
Study 53	☺	☺	?	☺	?	☺	☺
Study 54	☺	☺	?	☺	☺	☺	☺
Study 55	☺	☺	?	☺	☺	☺	☺
Study 56	☺	☺	☺	☺	☹	☺	☺
Study 57	☺	?	?	☺	☺	☺	☺

☺Low Risk ☹High Risk ? Unclear Risk

Table S4 Summarized LR and AUC of initial tests for CRC screening including or excluding the Shanghai CRC screening program

	No. of studies	Pooled values (95%CI)					No. of studies	Pooled values including the Shanghai program(95%CI)				
		LR^+ (95%CI)	I^2	LR^- (95%CI)	I^2	AUC (95%CI)		LR^+ (95%CI)	I^2	LR^- (95%CI)	I^2	AUC (95%CI)
Overall	103	9.56(7.91,11.47)	99.8	0.29(0.25,0.34)	99.7	0.86(0.81,0.92)	117	8.97(7.53,10.62)	99.9	0.33(0.28,0.38)	99.9	0.84(0.80,0.89)
Population												
Chinese	28	6.07(3.78,9.35)	99.9	0.49(0.38,0.68)	99.9	0.75(0.63,0.92)	42	5.64(4.09,7.63)	99.9	0.54(0.44,0.63)	99.9	0.74(0.67,0.83)
Other Asian populations	40	6.87(4.85,9.52)	99.8	0.43(0.35,0.52)	99.8	0.79(0.69,0.92)	54	6.32(4.84,8.15)	100.0	0.49(0.41,0.57)	99.9	0.77(0.70,0.83)
Western populations	63	11.65(9.53,14.14)	89.2	0.21(0.18,0.24)	99.8	0.94(0.93,0.92)	63	11.65(9.53,14.14)	99.6	0.21(0.18,0.24)	96.6	0.94(0.93,0.92)
Type of screening												
Organized	67	11.66(9.46,14.23)	99.7	0.28(0.23,0.34)	99.5	0.86(0.78,0.95)	81	10.29(8.44,12.43)	99.6	0.34(0.26,0.42)	99.6	0.83(0.77,0.90)
Opportunistic	36	6.56(4.63,9.10)	98.2	0.33(0.28,0.38)	76.6	0.89(0.87,0.92)	36	6.56(4.64,9.10)	98.2	0.33(0.28,0.38)	76.6	0.89(0.87,0.92)
Wave of screening												
First	48	11.66(9.02,14.84)	99.8	0.30(0.23,0.38)	99.5	0.84(0.74,0.97)	55	10.79(8.45,13.57)	99.9	0.34(0.26,0.42)	99.5	0.83(0.75,0.93)
Subsequent	19	11.86(8.13,16.84)	99.6	0.24(0.18,0.32)	99.0	0.91(0.88,0.94)	26	9.43(6.66,13.04)	99.9	0.35(0.24,0.47)	99.7	0.84(0.76,0.94)
Type of initial test												
FIT only	92	10.83(9.14,12.77)	99.6	0.26 (0.23,0.30)	99.3	0.90(0.88,0.92)	7	10.50(8.89,12.32)	99.0	0.27(0.23,0.31)	98.9	0.89(0.87,0.92)
RA only	5	2.29(0.76, 5.95)	98.4	0.79 (0.52,1.17)	97.9	0.60(0.33,1.00)	96	1.96(0.94,3.85)	99.9	0.84(0.69,1.03)	99.3	0.60(0.42,1.00)
RA & FIT (parallel)	3	2.07(0.77, 4.96)	99.9	0.50 (0.09,1.35)	95.6	0.92(0.71,1.00)	7	2.60(1.58,4.19)	99.7	0.39(0.25,0.58)	97.6	0.84(0.80,0.88)
RA & FIT (serial)	3	4.19(1.46,9.61)	99.3	0.91 (0.87,0.97)	94.3	0.69(0.56,0.90)	7	5.13(3.25,7.74)	98.5	0.91(0.89,0.93)	91.9	0.66(0.59,0.74)
No. of specimen for FIT												
1-specimen	66	9.73(7.82,11.98)	99.6	0.26 (0.22,0.31)	99.0	0.89(0.87,0.92)	68	9.58(7.75,11.74)	99.8	0.27(0.23,0.32)	99.4	0.89(0.86,0.91)
2-specimen	13	13.09(8.83,18.71)	99.6	0.26 (0.14,0.41)	99.1	0.90(0.79,1.00)	14	11.22(7.56,16.05)	99.8	0.28(0.17,0.41)	99.1	0.88(0.81,0.97)
Type of FIT												
Quantitative	66	9.70(7.80,11.96)	99.4	0.25 (0.21,0.29)	97.3	0.91(0.90,0.93)	66	9.70(7.80,11.96)	99.8	0.25(0.21,0.29)	99.6	0.91(0.90,0.93)
Qualitative	25	14.60(11.89,17.63)	99.8	0.28 (0.21,0.36)	99.6	0.89(0.81,0.97)	29	12.73(10.17,15.66	99.9	0.30(0.23,0.37)	99.5	0.87(0.82,0.93)
Cut-off for FIT (μg/g)												
<20	26	7.56 (5.94,9.54)	99.2	0.20 (0.15,0.24)	77.0	0.94(0.93,0.95)	26	7.56(5.94,9.53)	99.2	0.20(0.15,0.24)	77.0	0.94(0.93,0.95)
20	32	13.61(11.11,16.50)	99.7	0.25 (0.19,0.32)	99.7	0.89(0.84,0.96)	36	12.27(9.96,14.94)	99.9	0.27(0.22,0.34)	99.7	0.88(0.84,0.93)
>20	28	12.17 (7.83,18.20)	99.3	0.30 (0.24,0.36)	93.1	0.90(0.88,0.93)	28	12.17(7.82,18.17)	99.3	0.30(0.24,0.36)	93.1	0.90(0.88,0.93)

Abbreviations: CI: confidence interval; LR^+: positive likelihood ratio; LR^-: negative likelihood ratio; AUC: area under the receiver operating characteristic curve; RA: risk assessment; FIT: fecal immunochemical test.

Table S5 Meta-regression on sensitivity and specificity of initial tests for CRC

Study-level variables	No. of studies	Sensitivity				Specificity			
		Univariate analyses			Multivariable analyses*	Univariate analysis			Multivariable analysis*
		RR (95% CI)	*Overall P value*	Heterogeneity explained (%)	Adjusted RR (95% CI)	RR (95% CI)	*Overall P value*	Heterogeneity explained (%)	Adjusted RR (95% CI)
Population			< 0.01	21.63			< 0.01	<0.01	
Chinese	42	1.00 (ref)			1.00 (ref)	1.00 (ref)			1.00 (ref)
Other Asian populations	54	1.24 (1.02, 1.49)			1.70 (1.00, 2.89)	1.05 (1.01, 1.09)			1.75 (1.06, 2.90)
Western populations	63	1.31 (1.17, 1.47)			2.82 (2.08, 3.83)	1.05 (1.03, 1.07)			1.51 (1.09, 2.09)
Year of screening			0.47	<0.01			<0.01	<0.01	
Before 2015	45	1.00 (ref)			1.00 (ref)	1.00 (ref)			1.00 (ref)
After 2015	72	0.96 (0.84, 1.08)			1.37 (1.03, 1.83)	0.94 (0.92, 0.96)			0.51 (0.38, 0.67)
Type of screening			0.30	0.33			<0.01	<0.01	
Organized	81	1.00 (ref)				1.00 (ref)			
Opportunistic	36	1.07 (0.94, 1.22)				0.95 (0.93, 0.97)			
Sample size			0.08	0.64			<0.01	<0.01	
<10000	56	1.00 (ref)				1.00 (ref)			1.00 (ref)
>10000	61	0.90 (0.80, 1.01)				1.07 (1.05, 1.09)			2.48 (1.69, 3.62)
Wave of screening			0.75	<0.01			0.26	<0.01	
First	55	1.00 (ref)				1.00 (ref)			
Subsequent	26	0.98 (0.85, 1.13)				1.01 (0.99, 1.04)			
Type of initial test			<0.01	82.90			<0.01	69.55	
RA only	7	1.00 (ref)			1.00 (ref)	1.00 (ref)			1.00 (ref)
FIT only	96	1.49 (1.33, 1.67)			4.85 (2.76, 8.50)	1.12 (1.10, 1.15)			2.27 (1.29, 3.99)
RA & FIT (parallel)	7	1.49 (1.28, 1.74)			7.50 (3.66, 15.38)	0.91 (0.88, 0.94)			0.55 (0.26, 1.17)
RA & FIT (serial)	7	0.82 (0.70, 0.95)			0.38 (0.19, 0.78)	1.20 (1.16, 1.23)			9.99 (4.66, 21.41)
No. of specimen for FIT			0.74	2.82			0.24	<0.01	
1-specimen	68	1.00 (ref)				1.00 (ref)			
2-specimen	14	0.95 (0.81, 1.12)				1.02 (0.99, 1.04)			
Type of FIT			0.02	27.65			0.07	<0.01	
Quantitative	66	1.00 (ref)				1.00 (ref)			
Qualitative	29	1.14 (1.03, 1.27)				0.98 (0.96, 1.00)			

Cut-off for FIT (μg/g)			0.02	1.01			<0.01	<0.01	
<20	26	1.00 (ref)			1.00 (ref)	1.00 (ref)			1.00 (ref)
20	36	0.89 (0.81, 0.97)			0.84 (0.56, 1.26)	1.05 (1.04, 1.07)			2.21 (1.46, 3.32)
>20	28	0.89 (0.81, 0.98)			0.61 (0.40, 0.94)	1.03 (1.01, 1.05)			1.99 (1.30, 3.05)

RA: risk assessment; FIT: fecal immunochemical test; * VIFs less than 10 for all variables in multivariable models, indicating no collinearity.

Table S6 Comparisons of the current and the previous meta-analysis

	Meta-analysis					
	Current study	Imperiale, 2019	Stonestreet, 2019	Meklin, 2020	Phuangrach, 2022	Lee, 2014
Database	MEDLINE, EMBASE, Web of Science, Cochrane Library	Ovid MEDLINE, PubMed, Embase, and the Cochrane Library	MEDLINE	MEDLINE via PubMed, Ovid Embase, the Cochrane Library, and the Web of Science	MEDLINE	MEDLINE (via Ovid), EMBASE, Database of Abstracts of Reviews of Effects, Health Technology Assessment Database, Cochrane Database of Systematic Reviews, and Cochrane Central Register of Controlled Trials
Period for study searching	Up to 2022/1/5	Up to 2018/10	2007/1/1-2018/9/25	2020/2/29	2010-2021	Up to 2013/8/31
Studies included	Observational				Observational	RCT or cohort studies
Populations	Average-risk	Average-risk	Average-risk			Average-risk
Reference	Follow-up or colonoscopy	Colonoscopy	Colonoscopy	Colonoscopy		Follow-up or colonoscopy
Remarks	Including both opportunistic and organized screening using any cut-offs points for FIT to obtain sufficient data.				Brand of FIT kits were restricted to OC-Sensor	Using the cutoff value or sample number most commonly used in current practice in the United States
No. of studies	92	31	9	24	36	19
FIT only						
Pooled sensitivity	0.76 (0.72,0.79)		0.69 (0.54,0.81)	0.86 (0.78,0.93)	0.72 (0.66,0.78)	0.79 (0.69,0.86)
Pooled specificity	0.93 (0.92,0.95)		0.94 (0.92,0.95)	0.85 (0.81,0.88)	0.90 (0.87,0.92)	0.94 (0.92,0.95)
Pooled LR+	10.58 (8.89,12.52)		12.2 (10.1-14.7)			13.10 (10.49,16.35)
Pooled LR-	0.26 (0.23,0.30)		0.33 (0.21,0.51)			0.23 (0.15,0.33)
Pooled PPV	5.26 (4.81,5.71)					

Pooled detection rate	3.01 (2.74,3.28)					
AUC	0.89		0.95	0.87		0.95
Threshold for FIT <20 μg/g						
Pooled sensitivity	0.83 (0.78,0.86)					0.86 (0.75,0.92)
Pooled specificity	0.89 (0.86,0.91)					0.91 (0.89,0.93)
Pooled LR^+	7.56 (5.94,9.54)					9.8 (7.7,12.5)
Pooled LR^-	0.20 (0.15,0.24)					0.16 (0.09,0.28)
Pooled PPV	5.28 (4.52,6.04)					
Pooled detection rate	5.91 (4.72,7.11)					
Threshold for FIT =20 μg/g						
Pooled sensitivity	0.76 (0.70,0.82)	0.75 (0.61,0.86)				
Pooled specificity	0.94 (0.93,0.95)	0.95 (0.92,0.96)				
Pooled LR^+	13.61 (11.11,16.50)	14.19 (8.16,22.95)				
Pooled LR^-	0.25 (0.19,0.32)	0.26 (0.15,0.42)				
Pooled PPV	4.44 (3.74,5.13)					
Pooled detection rate	2.30 (1.92,2.67)					
Threshold for FIT>20 μg/g						
Pooled sensitivity	0.72 (0.66,0.78)	0.71 (0.56,0.83)				
Pooled specificity	0.94 (0.91,0.96)	0.95 (0.94,0.96)				
Pooled LR^+	12.17 (7.83,18.20)	15.49 (9.82,22.49)				
Pooled LR^-	0.30 (0.24,0.36)	0.30 (0.18,0.47)				
Pooled PPV	7.16 (6.30,8.03)					
Pooled detection rate	3.50 (2.70,4.30)					

Abbreviations: CI: confidence interval; LR^+: positive likelihood ratio; LR^-: negative likelihood ratio; PPV: positive predictive value; AUC: area under the receiver operating characteristic curve; RA: risk assessment; FIT: fecal immunochemical test.

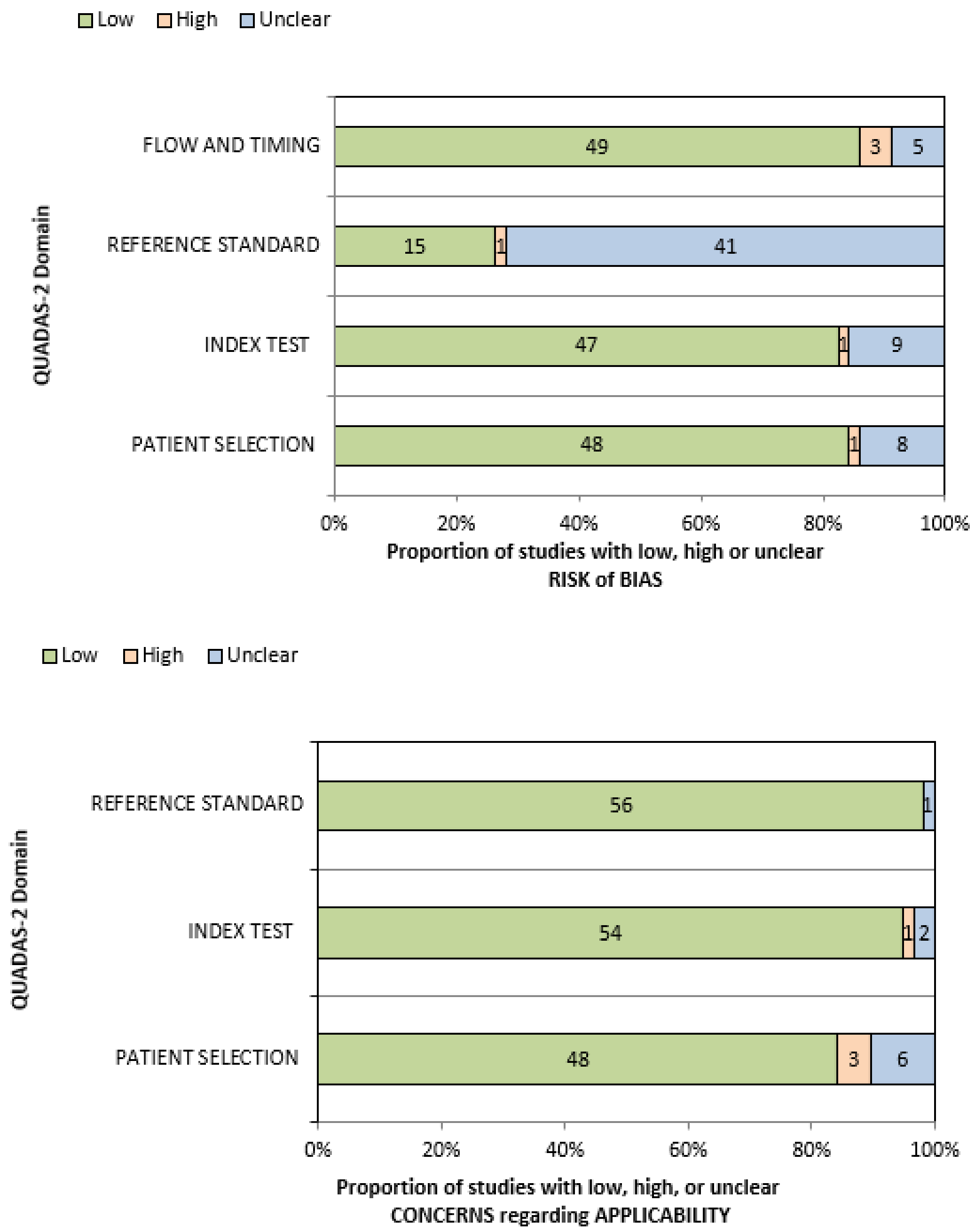

Figure S1. Risk of bias and applicability concerns graph: review authors' judgments for Risk of bias and applicability.

* Number in the bars indicating the number of studies with risks or concerns

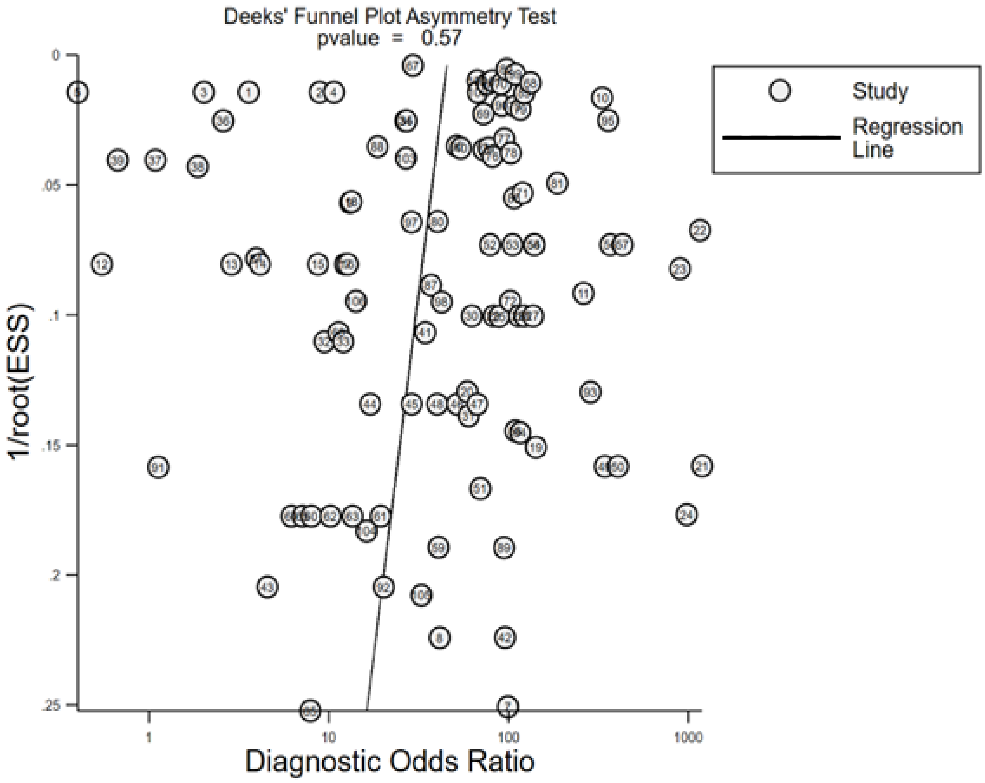

Figure S2. The Deek’s funnel-plot

P-value for publication bias presented in the figure.

Subgroups	No. of studies	Sensitivity (95% CI)	I^2	Specificity (95% CI)	I^2	Detection Rate (95% CI)	I^2	PPV (95% CI)	I^2
Overall	88	0.69(0.63,0.74)	99.8	0.93(0.92,0.94)	100.0	2.35(2.14,2.56)	99.7	3.43(3.20,3.67)	99.8
Population									
Chinese	24	0.40(0.29,0.53)	99.9	0.93(0.89,0.95)	100.0	1.08(0.85,1.32)	99.8	1.46(1.24,1.68)	99.8
Other Asian populations	41	0.50(0.41,0.59)	99.8	0.93(0.91,0.95)	100.0	1.37(1.13,1.60)	99.8	1.99(1.75,2.23)	99.8
Western populations	47	0.81(0.77,0.85)	91.8	0.94(0.92,0.95)	99.9	3.55(3.05,4.04)	99.0	5.16(4.56,5.75)	97.7
Type of screening									
Organized	62	0.66(0.59,0.74)	97.9	0.94(0.92,0.95)	100.0	2.08(1.86,2.30)	99.8	3.21(2.96,3.46)	99.8
Oppotunistic	26	0.75(0.68,0.80)	99.4	0.91(0.88,0.94)	100.0	5.02(4.10,5.95)	69.8	6.33(4.74,7.92)	90.1
Wave of screening									
First	40	0.66(0.56,0.75)	99.5	0.93(0.92,0.95)	100.0	2.17(1.89,2.44)	99.8	3.32(2.99,3.66)	99.9
Subsequent	22	0.68(0.55,0.78)	99.2	0.94(0.91,0.96)	100.0	1.95(1.53,2.37)	99.8	3.06(2.65,3.48)	99.8
Type of initial test									
RA only	5	0.35(0.19,0.56)	99.6	0.75(0.58,0.87)	100.0	0.62(0.50,0.73)	89.2	0.53(0.26,0.80)	99.5
FIT only	71	0.75(0.71,0.79)	99.5	0.94(0.93,0.94)	100.0	2.79(2.53,3.04)	99.3	4.48(4.09,4.86)	99.6
FIT and RA (Parallel)	5	0.76(0.63,0.86)	99.7	0.67(0.40,0.86)	100.0	1.84(1.69,2.00)	93.5	0.83(0.74,0.92)	96.2
FIT and RA (Serial)	7	0.12(0.10,0.13)	60.5	0.98(0.97,0.99)	98.7	0.28(0.24,0.31)	87.7	1.51(0.92,2.09)	98.9
No. of FIT									
1 sample FIT	49	0.76(0.71,0.81)	98.8	0.93(0.92,0.94)	100.0	3.57(3.18,3.95)	99.4	5.17(4.61,5.74)	99.6
2 sample FIT	9	0.70(0.49,0.84)	93.5	0.94(0.91,0.96)	100.0	1.65(1.10,2.20)	99.3	1.99(1.65,2.34)	97.2
Type of FIT									
Quantitative	45	0.78(0.73,0.82)	96.3	0.93(0.92,0.94)	99.9	3.94(3.49,4.40)	98.6	5.44(4.97,5.90)	95.8
Qualitative	19	0.73(0.63,0.81)	99.4	0.97(0.94,0.98)	100.0	1.68(1.36,2.00)	99.9	3.60(2.76,4.44)	99.7
Cut−off for FIT (µg/g)									
<20	19	0.81(0.75,0.86)	98.0	0.90(0.88,0.92)	99.7	5.51(4.11,6.92)	96.5	5.17(4.29,6.04)	90.8
20	30	0.74(0.67,0.80)	99.6	0.94(0.93,0.95)	100.0	2.10(1.79,2.41)	99.6	3.75(3.27,4.23)	99.8
>20	17	0.74(0.64,0.82)	90.3	0.96(0.95,0.96)	99.6	3.24(2.31,4.17)	97.4	6.59(5.61,7.56)	83.4

Figure S3. Sensitivity analysis on pooled sensitivity, specificity, detection rate and AUC of initial screening tests by study characteristics.

Abbreviations: CI: confidence interval; PPV: positive predictive value; RA: risk assessment; FIT: fecal immunochemical test.

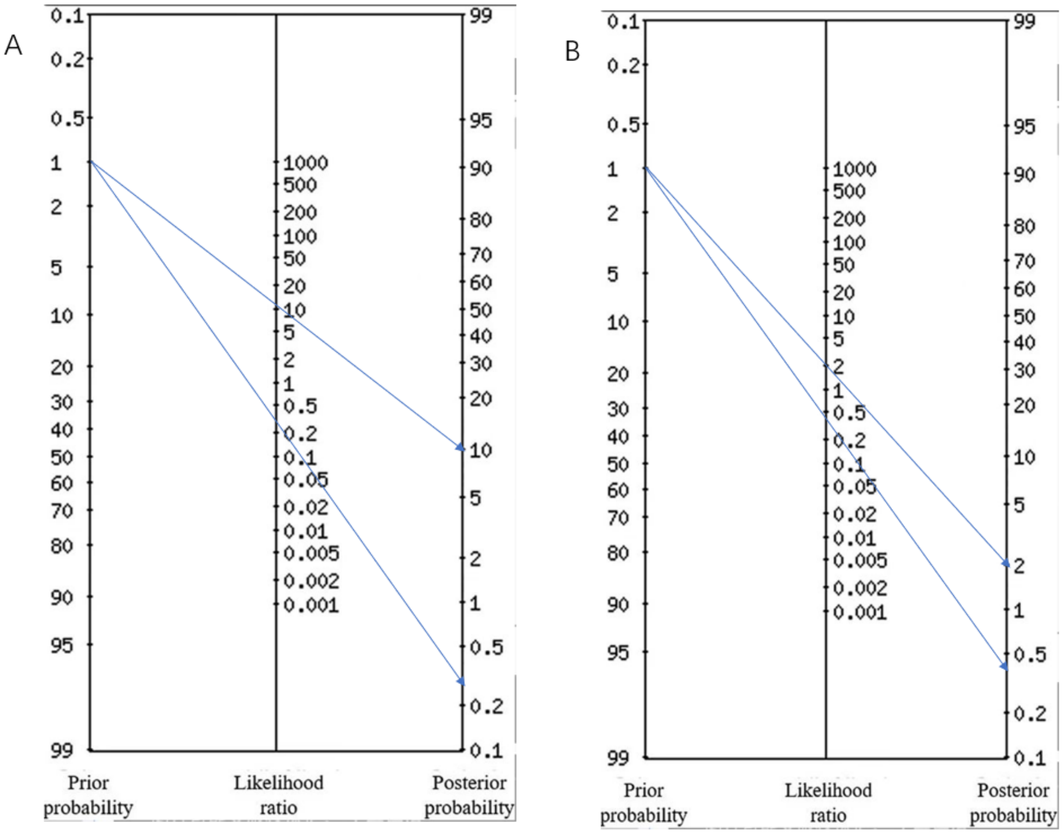

Figure S4. Fagan's nomogram for FIT only (A) and parallel use of RA and FIT (B)

*The prior probability set at 1%, with LR^+ of 10.45 and LR^- of 0.27 for FIT only, and the post probability of 10.45% and 0.27%; or with LR^+ of 1.96 and LR^- of 0.41 for parallel use of RA and FIT, and the post probability of 1.96% and 0.41%

Abbreviations: LR^+: positive likelihood ratio; LR^-: negative likelihood ratio; RA: risk assessment; FIT: fecal immunochemical test.
